# National Safety Associates nutritional supplementation trial of fruit and vegetable extracts and vascular function (NNTV): study protocol for a randomised controlled trial

**DOI:** 10.1186/s13063-016-1185-8

**Published:** 2016-02-04

**Authors:** Marietta Sayegh, Melina Tsiountsioura, Polly Page, Dan Del Rio, Sumantra Ray

**Affiliations:** Medical Research Council (MRC) Human Nutrition Research Unit, Cambridge, UK; The Need for Nutrition Education/Innovation Programme (NNEdPro), University of Cambridge, Cambridge, UK; Department of Food Science, University of Parma, Parma, Italy

**Keywords:** Vascular function, endothelial function, fruit and vegetable extracts, capsule supplementation, placebo, carotid intima media thickness, double-blind RCT

## Abstract

**Background:**

Cardiovascular disease has a multifactorial aetiology with a number of both modifiable and non-modifiable risk factors. Although evidence indicates that dietary intake plays an important role, few studies have focused on the effect of fruit and vegetable consumption on early markers of vascular function. Therefore, we hypothesised that supplementation with capsules containing a combination of fruit and vegetable extracts over 12 weeks can significantly modulate biomarkers of vascular function compared with a control group receiving placebo.

**Methods/Design:**

This is a double-blind, randomised controlled trial that includes overweight and obese but otherwise healthy participants. Participants are randomly allocated to one of two groups: active supplementation (encapsulated fruit and vegetable powder) or placebo taken twice daily for 12 weeks, whereas both groups will be given the ‘5-A-Day’ dietary advice. The primary outcome is to measure changes to the carotid intima media thickness (cIMT) between the two groups from baseline (test visit 1) to 12 weeks later (test visit 2). The secondary outcomes include macro- and microvascular changes and changes to blood markers.

**Discussion:**

In addition to the primary and secondary objectives, this explanatory trial incorporates potential novel biomarkers such as trimethylamine-N-oxide (TMAO) and lipopolysaccharide (LPS).

**Trial registration:**

ISRCTN14315618. Registration date 27 February 2014.

## Background

### Background and rationale

Increasingly, dietary intake and nutrition are recognised as playing a major role in the aetiology of CVD [1]. A number of epidemiologic and observational studies regularly report that consuming fruit and vegetables (F&Vs) can lower CVD risk. Among several studies that have looked into the beneficial effects of F&Vs in lowering CVD risk, Yusuf and colleagues [2] reported that the population attributable risk for acute myocardial infarction given irregular consumption of F&Vs was 12.9 %. Also, Crowe and colleagues [3] reported that participants consuming at least 80 g of F&Vs per day had a 22 % lower risk of fatal ischaemic heart disease. Berry fruits have also been shown a reduction in blood pressure as well as a reduction in the levels of pro-inflammatory markers and adhesion molecules, which are both markers of endothelial function [4].

Endothelial dysfunction is emerging as a potential set of early markers of cardiovascular disease risk. It is considered to play a principal role in the initiation and progression of atherosclerosis, and it is also present in later stages of vascular disease. Many studies have used a variety of tests in order to measure endothelial function as well as to assess the impact of potentially therapeutic interventions on the vasculature [5]. In a placebo-controlled crossover study, a short fruit extract intervention study led to improved endothelial vasodilation measured by flow mediated dilatation (FMD) in individuals at risk for CVD [6].

Evidence suggests that dietary flavonoids work in various ways and reduce oxidative stress, which is caused by factors such as smoking, obesity, hypertension and other pathological conditions [7]. Specifically, phytonutrients in F&Vs may have specific cardio-protective effects partially mediated through favourable effects on endothelial function, inhibition of angiogenesis and cell migration and proliferation in blood vessels [8]. Although recent health and public reports focus on the five portions of a range of F&V as being cardio-protective [9, 10], clear dose-response relations are poorly defined, which has resulted in major inconsistencies in F&Vs recommendations. Overall, the body of evidence is growing on the modulation of endothelial, as well as macro- and microvascular function (predictive of cardiovascular risk), due to interventions using different categories of F&Vs or chronic ingestion of selected phytonutrients. However, a paucity of studies have examined the vascular modulatory effects of chronic unselective exposure to a high dose of a very wide range of phytonutrients derived from F&Vs, and it is currently not known whether this can bring about similar magnitudes and patterns of vascular change as compared with exposure to selected F&Vs. We hypothesize that a high dose of unselective phytonutrients derived from F&Vs, when ingested chronically, will result in potentially beneficial changes (compared with placebo) to both macro- and microvascular measures, through modulation of endothelial and conduit function of arteries, thus mitigating cardiovascular risk.

## Methods/Design

### Aim/Objectives

The aim of this study is to evaluate the effects of supplementation with encapsulated fruit and vegetable powder compared with placebo on vascular and endothelial function before and after 12 weeks of intervention when they are both given with ‘5-A-Day’ dietary advice verbally and in writing.

Specifically, the primary objective of this trial is to assess whether there is a change in the carotid intima media thickness (cIMT) between the two groups.

The secondary objectives are to assess the following:

1. Dynamic macrovascular and microvascular changes.
2. Changes in laboratory blood markers, including endothelial cell function and oxidative stress.
3. Changes in Knowledge, Attitudes and Practices (KAP) using the Five-a-day Community Evaluation Tool (FACET).
4. Changes to safety indicators: liver function, routine bloods (including full blood count) and ECG.

### Study design

This study is a double-blind, randomised controlled trial for overweight and obese but otherwise healthy participants. It is being conducted at the Medical Research Council (MRC), Human Nutrition Research (HNR) unit in Cambridge, United Kingdom. The study had two stages of ethical approval; first, an internal research governance approval was granted by the Research Review Board (RRB) at MRC HNR, which comprises joint scientific and ethical peer review. Full independent research ethics approval was subsequently obtained from the NHS National Research Ethics Service (REC Reference: 13/EE/0095) and fully informed written consent is taken from all participants. Confirmation that this study does not fall under the legal definition of a Clinical Trial of an Investigational Medicinal Product (CTIMP) was sought from the Medicines and Healthcare Products Regulatory Agency (MHRA) Borderline Team.

### Participants and setting

#### Inclusion criteria

Participants are eligible for this study if they fulfil the following criteria: (1) aged between 25 and 65 years; (2) BMI 25 to 35 kg/m^2^; (3) able and willing to take gelatin casing capsule supplements for 12 weeks and complete the required evaluations; and (4) competent and willing to give informed consent.

#### Exclusion criteria

People with any of the following will be excluded from participation: (1) all diagnosed cardiovascular risk factors or disorders; (2) diabetes and disorders of glycaemic control; (3) irritable and inflammatory bowel disorders and acid peptic disease; (4) any active tumours/cancers; (5) lipid or cholesterol lowering tablets; (6) continuous use of any nutritional supplements and/or prescription medication likely to impact on study measurements or safety; (7) pregnancy or breastfeeding; (8) high-dose aspirin and analogues; (9) high levels of physical activity; (10) current active mental and/or neurological illness and the use of related medication; and/or (11) currently smoking.

### Sample size

The sample size calculation was performed according to cIMT changes over 12 weeks of supplementation. cIMT normal values in the general adult population (including overweight individuals) less than 65 years of age, range from 0.60 to 0.80 mm with a SD of 0.10 mm. [7]. Assuming a two-tailed t-test using a 5 % significance level (two-sided), a sample size of 37 in each group (74 participants in total) was determined to have 85 % power to detect a mean cIMT difference of 0.07 mm after 12 weeks of supplementation between the intervention and control groups, assuming a common SD of 0.10 mm. Allowing for a dropout rate of approximately 15 %, we aim to recruit 80 participants in total; *n* = 40 in the placebo and *n* = 40 in the intervention group.

### Randomisation and blinding

When all inclusion criteria have been met and informed consent has been obtained, participants will be randomly allocated to one of the two groups. Randomisation is based on a blocked randomisation sequence generated by a research statistician independent to the study team and with the sequence unknown to both researchers and participants. Two independent code keepers at MRC HNR, who are not involved in the running of the study, are securely holding the randomisation code. To maintain scientific integrity, at no point will those either generating or holding the study codes, come into contact with participants or data analyses. The details of the allocation are concealed from the research team and the participants until all data collection and analysis are complete. Blinding of participants and research team to allocation status will be assured by identical capsule appearance and identical labelling between the placebo and the active supplementation. Blinding of the capsules, packaging and labelling have been undertaken by the funding company (National Safety Associates (NSA) LLC, USA).

#### Capsule supplementation

Participants are asked to take six 00 capsules daily: three capsules twice with meals and plenty of water. The intervention supplements were made by blending three separate formulas. Two of these formulas contain a dried powder blend of juice and pulp of a number of fruits like apple, grape and blueberry, whereas the last one consists of vegetable juice powder and pulp from different vegetables such as carrot and broccoli. The total amount of nutrients contained per six capsules can be found in Table 1. The placebo capsules contain microcrystalline cellulose, dicalcium phosphate, magnesium stearate and FD&C yellow #6.

**Table 1:** Intervention supplement nutritional value (per six capsules)

Nutrient	Active
B-carotene	1.8 mg
Vitamin E	22 mg
Vitamin C	300 mg
Folate	200 μg

### Study procedures

#### Recruitment and pre-screening questionnaire

**Fig. 1:**
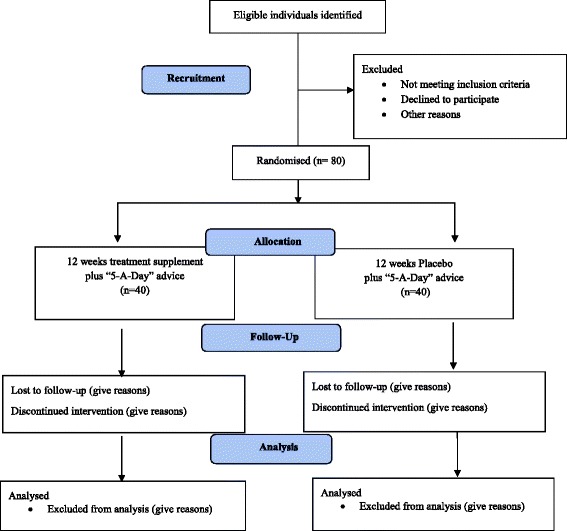
CONSORT flow diagram

Figure 1 summarises the National Safety Associates nutritional supplementation trial of fruit and vegetable extracts and vascular function (NNTV) recruitment and participant follow-up plan. Table 2 provides an overview of the study procedures performed at each visit.

**Table 2:** Schedule of samples and data collection

Task	Pre-screening (telephone)	Screening	Test visit 1 (week 0)	Intermediate visit (week 6)	Test visit 2 (week 12)
Pre-screening questionnaire	X				
Obtain informed consent		X			
Study eligibility/screening	X	X			
Medical history		X			
Height, weight and BMI	X	X	X		X
Waist circumference			X		X
Body composition (bioelectrical impedance)			X		X
Spot urine sample (10 ml) for hydration status assessment including osmometry and including dipstick			X		X
24-hour urine collection			X		X
Physical activity questionnaire (IPAQ)		X	X		X
Standardised ‘5-A-Day’ advice		X	X	X	X
Group randomisation			X		
FACET (‘5-A-Day’/Lifestyle) Questionnaire		X	X	X	X
Blood pressure (standard automated at screening; seated, supine and ambulatory in TV1 and TV2)		X	X		X
Pulse wave analysis			X		X
Carotid intima-media thickness			X		X
12-lead ECG			X		X
and GTN-induced dilatation			X		X
Laser doppler iontophoresis			X		X
Assess continuance criteria			X	X	X
Blood samples		X	X	X	X
Assess concomitant medication	X	X	X	X	X
Test product dispensed			X	X	
Compliance assessment				X	X
Tablet count				X	X
Assess adverse reactions				X	X
Empty containers collected				X	X
Collect unused test product					X

Participants are recruited following one of the following methods: they are either identified from the MRC HNR volunteer database and are sent a letter inviting them to participate in the study, or they respond to a study advert placed around the community and in local magazines. Once the volunteer expresses initial interest in participating in the study, he/she is contacted via telephone by the study coordinator or research assistant. Both the participant information sheet (PIS), along with a leaflet describing all the vascular measurements used in the study, are sent to the potential participant, allowing them at least 24 hours to read and understand the study aim and procedures and decide if they would like to take part in the study.

The pre-screening telephone call lasts approximately 15 minutes and follows the structure of a standardised pre-screening questionnaire. The questionnaire assesses the potential eligibility of the participant for the study through a series of questions around the inclusion/exclusion criteria (including medication and self-reported height and weight) for the study.

Potential participants who are found not to be eligible will be excluded at this stage. Eligible participants will be invited to attend the study site (MRC HNR) for a further screening visit.

#### Screening visit

Following completion of the pre-screening questionnaire and provided that the participant is eligible to take part in the study, he/she is asked to attend the screening visit, which takes place at HNR and lasts approximately 2 hours.

Informed consent is obtained from all participants during the screening visit, and any questions relating to the study are answered, ensuring that the participant has fully understood all the study procedures before continuing. Consent is obtained before any measurements are taken. Height is measured to the nearest 0.1 cm using a stadiometer, and weight to the nearest 0.1 kg using calibrated mass scales. BMI is calculated using these measurements. Two blood pressure readings are recorded while the patient is in a seated position. A third BP measurement is performed if the first two readings have a discrepancy of more than 10 mmHg.

A full eligibility assessment is carried out using the HNR volunteer health screening and demographics questionnaire. Medical history and any recent changes in medication since the pre-screening questionnaire are also assessed and evaluated at this stage. In addition, female participants are also asked about the timing of their menstrual cycle as this can affect the vascular measurements in the study. Fasting blood samples are collected by venepuncture to measure blood lipids, glucose, and liver function as a routine safety measure and as an indicator of normal metabolism. Table 3 summarises all the blood markers that are examined during the study. At the end of the visit, the volunteers are asked to complete two short questionnaires: the International Physical Activity Questionnaire (IPAQ) and the Five-a-day Community Evaluation Tool (FACET) and are provided with ‘5-A-Day’ dietary advice. If successfully screened, the participant is invited back to complete test visits 1 and 2. Ineligible participants are thanked for their time and are offered to join the HNR volunteer database. Breakfast is provided to all potential volunteers before the end of the visit.

**Table 3:** Blood parameters analysed in the National Safety Associates nutritional supplementation trial of fruit and vegetable extracts and vascular function (NNTV) study

	Biological parameter	Screening visit	Test visit 1 (week 0)	Intermediate visit (week 6)	Test visit 2 (week 12)
Screening/Safety and Research	FBC	X	X	-	X
	HbA1c	X	X	-	X
	HDL	X	X	-	X
	LDL	X	X	-	X
	Total cholesterol	X	X	-	X
	Liver function (ALT)	X	X	-	X
	Creatinine	X	X	-	X
	Fasting glucose	X	X	-	X
	Potassium	X	X	-	X
Research	Oxidized LDL		X	-	X
	C-Peptide		X	-	X
	Fasting insulin		X	-	X
	*Apo* A1		X	-	X
	*Apo* A2		X	-	X
	*Apo* B		X	-	X
	*Apo* E		X	-	X
	Cholesterol esters		X	-	X
	Oxysterols		X	-	X
	Isoprostanes		X	-	X
	P-Selectin		X	-	X
	E-Selectin		X	-	X
	IgM phosphorylcholine		X	-	X
	Vitamin A		X	X	X
	Vitamin C		X	X	X
	Vitamin E		X	X	X
	Carotenoids		X	X	X
	TMAO^d^		X	-	X
	LPS^e^		X	-	X
	DNA, Metabolomics and other relevant stored sample analyses		X	-	X
	Urine		X^a, b^	-	X^a, b^

(1). All screening bloods will be analysed at the Clinical Biochemistry and Immunology Laboratory at Addenbrooke’s Hospital. (2). Participants may be excluded from the study if screening/safety blood results are deranged. Spot urine collection: ^a^24 hour urine collection: ^b^(3) Additional 15-ml blood samples will be collected at test visit 1 for future DNA analysis and at test visit 2 for open profile metabolomics. FBC, full blood count; HDL, high density lipoprotein; LDL, low density lipoprotein; ^d^TMAO, trimethylamine-N-oxide; ^e^LPS, lipopolysaccharide

#### Pre-test visits 1 and 2

Participants are asked to collect their urine for 24 hours prior to test visits 1 and 2. Participant randomisation takes place prior to test visit 1.

#### Test visit 1

Test visit 1 lasts approximately 5 hours. Any changes to medication or health status are re-assessed in order to evaluate whether participants are still suitable to participate in the study. Height and weight are also measured as previously described, and BMI is calculated from these measures. Three waist circumference readings are taken, measured to the closest 0.1 cm with an anthropometric measuring tape. Body fat percentage, as well as total body water, are calculated using a four-point bioelectrical impedance Tanita BC-418.

Three blood pressure readings are measured in seated and supine positions before starting the cardiovascular measurements. Blood pressure is also measured using the ambulatory blood pressure (ABP) device, which is programmed to record readings every 15 minutes. In addition, fasting blood samples are collected during the visit to measure the blood markers mentioned in Table 3, including fasting blood glucose, as well as HbA1c. The following cardiovascular measurements with the following sequence are performed in a fasting state: carotid intima media thickness (cIMT), pulse wave analysis (PWA) using sphygmocor applanation tonometry, laser Doppler iontophoresis (LDI), 12-lead ECG, flow-mediated dilatation (FMD) and glyceryl trinitrate-induced vasodilation. In addition, participants provide a single urine sample, and a member of the study team will carry out a hydration check using an osmometer and urine dip stick in order to measure hydration status. Fluid intake is also monitored during the test visits 1 and 2. At the end of the visit, participants complete the IPAQ and FACET questionnaires and are provided with ‘5-A-Day’ dietary advice. Before participants leave, they are issued with 6 weeks supply of capsules. Breakfast/lunch is provided to all participants at the end of the visit.

#### Intermediate visit (6 weeks)

The intermediate visit takes place 6 weeks after test visit 1 and lasts approximately 1 hour.

During this visit, a member of the study team assesses any changes to medications or health status to ensure that participants are still suitable for the study. Participants return the batch of capsules given during test visit 1 and are given the next 6 weeks supply of capsules. Remaining capsules are counted in order to ensure compliance with the protocol. In addition, fasting blood samples are collected to further monitor compliance with the intervention by testing vitamins A, C and E and carotenoid levels. At the end of the visit, participants complete the FACET questionnaire and are also provided with the ‘5-A-Day’ dietary advice. Breakfast is provided at the end of the visit.

#### Test Visit 2

This visit is identical to test visit 1 except that participants are not provided with more capsules. At the end of the visit, participants are thanked for their time and given forms for reimbursement.

### Analytic vascular measurements procedures

#### Carotid intima media thickness (cIMT)

cIMT is performed using a B-mode ultrasound imaging of the right and left carotid arteries with 7.5 MHz linear array transducer. In a dark, quiet room, participants are in a supine position with their head turned 45 ^o^ in the direction opposite to the carotid artery being measured. Eight longitudinal images of the distal common carotid arteries (CCA) are obtained, four from each side. On completion of the measurements, the images are analysed by two members of the research team. The outcome variable is the mean CCA-IMT defined as the average of four measurements of the far wall IMT of the right and left CCA.

#### Pulse wave analysis (PWA)

Aortic augmentation index (AIx) is derived from the right wrist (radial pulse) using the SphygmoCor Vx version 7.01 (AtCor Medical, Sydney, Australia). The SphygmoCor device provides a quality index (QI), which represents reproducibility of the waveform, and any recordings with a QI < 80 are excluded. Because the AIx changes with heart rate, the software adjusts this value for a heart rate of 75 beats per minute (AIx@HR75).

#### Laser Doppler imaging following iontophoresis

Vascular changes related to microcirculatory flow and endothelial function are identified using the laser doppler imaging (LDI) technique (Moor Instruments, UK) with iontophoresis of acetylcholine (Ach) and sodium nitroprusside (SNP). The room temperature is set at 22 to 23 °C, and the measurement is taken from the participant’s right arm while he/she is in a seated position. Each drug is administered at increasing currents of 0 μA, 40 μA, 60 μA and 100 μA for 5, 10, 30 and 60 seconds, respectively. Cumulative dose response curves are analysed automatically by the built-in software (MoorVMS-PC software).

#### Electrocardiogram (ECG)

A 12-lead ECG is recorded in each participant with a 12-channel ECG machine (GEM MED, GEM heart one+, Cambridge, UK) on a paper with a speed of 25 mm/sec and 10 mm/mv standardisation. ECG waveforms are also saved in the gem heart viewer software (GEM MED, Cambridge, UK) for further examination and analysis if needed. ECG is performed once during test visits 1 and 2.

#### Flow-mediated dilatation and induced vasodilation using glycerol trinitrate

FMD is assessed in the brachial artery by high-resolution ultrasound and computerised edge detection system (FMD studio, Quipu srl, Pisa, Italy). All FMD measurements are performed while the participants are in a supine position with their right arm extended and in a quiet, darkened room kept at a relatively constant temperature (22 to 23 °C) to minimise the possible negative effect of environmental and physiological influences. The blood pressure cuff is placed on the right forearm, 1 to 2 cm distal to the elbow crease, and an ultrasound probe holder is used to hold the probe stable on the antecubital fossa. The brachial artery is continuously imaged using a 7.5-MHz probe in B-mode (Accuson X300, Siemens UK; with a VF 8-3 probe) at a depth of 3 cm. The brachial artery diameter is measured on acquired frames using Quipu software (Quipu srl, Pisa, Italy). Baseline brachial artery images and blood-flow velocity prior to occlusion cuff inflation are recorded for 1 minute. The occlusion cuff is then inflated to 200 mmHg for 5 minutes and released, thereby inducing reactive hyperaemia and endothelium-dependent vasorelaxation, which is monitored for 4 minutes. Following a 10-minute recovery period, endothelium independent dilation is monitored over a 5-minute period after sublingual administration of 300 μg glycerol trinitrate (GTN) spray. Flow-mediated and GTN-induced dilatation of the brachial artery are expressed as maximum percent dilatation following cuff deflation and GTN administration, respectively.

### Side effects and safety assessments

All participants are provided with a booklet where they can record any potential side effects. In case they develop severe side effects, they are advised to stop the supplementation and will be excluded from the study. All serious and non-serious adverse events and/or reactions involving capsules supplementation are logged by a member of the study team in a password protected database. In addition, any abnormal results of clinical importance detected during the course of the study (screening, test visits 1 and 2) will be reported by the HNR clinical team to the participant’s general practitioner (GP).

### Biological sampling

A 15-ml blood sample will be collected from each participant at the screening visit and approximately 40 ml will be collected during test visits 1 and 2. Table 3 outlines the blood parameters that are analysed in each study visit.

Participants collect-24 hour urine samples, which will be used to investigate the bioavailability of key polyphenol compounds present in the capsules. In addition, hydration status is measured using urine osmolality and urine dip stick from both the urine collected over 24 hours and from the single urine sample collected during test visits 1 and 2.

### Data analysis

A consort flowchart of enrolled participants will be provided, and the demographic characteristics recorded at the time of randomisation will also be described. Descriptive analysis will be conducted for all baseline variables to compare participants in both study groups. Results will be reported as means and standard deviations for continuous variables and frequencies with percentages for categorical variables. Two group comparisons for changes in primary and secondary outcome measures following 12 weeks of intervention will be made, assuming similar levels of variance between the two treatment groups and using two-tailed t-tests for normally distributed data. Paired t-tests will be used to assess within-group changes in study outcomes. Multiple linear regression models will be used to assess whether changes from baseline to 12 weeks were significant between the study groups. Regression analysis will be conducted, with adjustment for potential baseline confounding variables. Additionally, a sensitivity analysis will be performed, exploring variation in the standard deviation between the differences of the mean change in cIMT measurements. Exploratory within-group changes will also be assessed for each outcome measure.

## Discussion

This study was designed to test the hypothesis that supplementation with capsules containing a combination of fruit and vegetables, in addition to dietary advice to consume five portions of fruit and vegetables daily, will significantly modulate biomarkers of vascular and metabolic function in overweight and obese adults, compared with a control group receiving identical dietary advice to consume ‘5-A-Day’ and placebo capsules. The particular population was chosen for this study as overweight and obese individuals have increased cytokines free fatty acids and inflammation markers. These increases can lead to early endothelial dysfunction due to increased inflammation and other mechanisms associated with overweight and obesity.

This study was powered with cIMT as a primary outcome. However, as cIMT is a dynamic but structural measurement, a number of functional measurements, including those assessing vascular endothelium and smooth muscle tone, as well as both factors together, form part of the panel of outcome measures used in this mechanistic trial. These measures were designed to provide novel insights into vascular function pathways and the prediction of cardiovascular risk. In addition to vascular measurements, waist circumference, blood pressure and hydration status are expected to provide complementary information.

In particular, this explanatory trial also incorporates potential novel biomarkers such as trimethylamine-N-oxide (TMAO) and lipopolysaccharide (LPS). Polyphenolic compounds have recently been found to modulate TMAO [11, 12]. Evaluating the ability of the NNTV study interventions on TMAO concentrations might be useful to understand if the polyphenolic fraction of this extract acts as a gut microbial modulator, reducing cardiovascular disease risk through the control of trimethylamine production.

Lipopolysaccharide (LPS), also known as endotoxin, is a component of the cell wall of gram-negative bacteria that may normally reside in the colon of humans. Additionally, polyphenolic compounds have been attributed to modulation of the gut microbiome, through mechanisms involving their bacteriostatic activity and the inhibition of microbial fermentation [11, 12]. Assessing the ability of the fruit and vegetable extract to change levels of LPS can help explain if the polyphenolic fraction of this extract acts as a gut microbial modulator, reducing future disease risk through the control of endotoxaemia.

### Trial Status

This clinical trial was registered in February 2014. At the time of manuscript submission, the enrolment of volunteers was ongoing. The estimated study completion date is December 2015. Please refer to this study by its ISRCTN identifier: ISRCTN14315618.

## Abbreviations

ACh: acetylcholine
BMI: body mass index
cIMT: carotid intima media thickness
CTIMP: clinical trial of an investigational medicinal product
CVD: cardiovascular disease
ECG: electrocardiogram
F&Vs: fruit and vegetables
FACET: Five-a-day Community Evaluation Tool
FMD: flow-mediated dilatation
GTN: glycerol trinitrate
HNR: Human Nutrition Research
IPAQ: International Physical Activity Questionnaire
KAP: knowledge, attitudes and practices
LDI: laser doppler iontophoresis
LPS: lipopolysaccharide
MHRA: Medicines and Healthcare Products Regulatory Agency
MRC: Medical Research Council
NNTV: NSA Nutritional Supplementation Trial of Fruit and Vegetable Extracts and Vascular Function
NSA: National Safety Associates
PIS: participant information sheet
PWA: pulse wave analysis
QI: quality index
RRC: research review board
SNP: sodium nitroprusside
TMAO: trimethylamine-N-oxide
